# A Manual of Auscultation and Percussion

**Published:** 1891-01

**Authors:** 


A MANUAL OF AUSCULTATION AND PERCUSSION, embracing the physical diagnosis of diseases of the lungs and heart, and of thoracic aneurism. By Austin Flint, M. D., L. L. D. Fifth edition thoroughly revised by J. C. Wilson, M. D., lecturer on physical diagnosis in the Jefferson Medical College &c., illustrated with wood cuts. Philadelphia; Lea Brothers & Co., 1890.

It is a genuine pleasure to go over the pages of Dr. Flint's manual on auscultation, &c., which has just appeared in its fifth edition. The work of revision was undertaken by a distinguished teacher in the Jefferson Medical College, Dr. J. C. Wilson, who we know to be equal to the task he has assumed. To undertake to review this book in detail would be a work of supererogation, as the author and his manual are too well known, sufficient to say that the manual has not lost by the revision, unlike a majority of books of such long standing, but has had inserted into it all that was considered new. Dr. Flint may be considered a pioneer in physical diagnosis of diseases of the lungs and heart. The principles incorporated in this manual have stood the test of time, thus showing how well he understood the field in which he labored for so many years. The crepitant rales which we were once taught to regard as significant of pneumonia, is now questioned as to whether it indicates pneumonia or pleurisy. We think post mortem should have settled this question long since.

This little book should be in the hands of every physician, it is a key to a clear understanding of the pathological conditions concealed in the walls of the chest; diagnosis and prognosis makes a physician. The book is neatly and substantially issued and is in keeping with the reputation, the publishing house of Lea Brothers & Co. have so deligently labored to establish. A. B. P.

